# Combined resection of a tumor and the inferior vena cava: report of two cases

**DOI:** 10.1007/s00595-012-0337-z

**Published:** 2012-09-22

**Authors:** Masatoshi Jibiki, Yoshinori Inoue, Toshifumi Kudo, Takahiro Toyofuku, Kazutaka Saito, Kazunori Kihara, Atsushi Kudo, Daisuke Ban, Shigeki Arii

**Affiliations:** 1 Department of Vascular Surgery, Tokyo Medical and Dental University, 1-5-45 Yushima, Bunkyo-ku, Tokyo, 113-8519 Japan; 2 Department of Urology, Tokyo Medical and Dental University, Tokyo, Japan; 3 Department of Hepato-Biliary-Pancreatic Surgery, Tokyo Medical and Dental University, Tokyo, Japan

**Keywords:** Inferior vena cava tumor thrombus, Inferior vena cava invasion, Renal cell carcinoma

## Abstract

Tumor resection and caval tumor thrombectomy, with or without cavotomy and inferior vena cava (IVC) replacement are sometimes performed in patients with renal cell carcinoma (RCC) extending into the IVC or liver tumors invading the IVC. Two such cases were treated. Case 1: a 68-year-old female was transferred with a diagnosis of right RCC with tumor thrombus extending into the IVC. A plication was performed to prevent extension into the right atrium before the nephrectomy and cavotomy with removal of the tumor thrombus was accomplished, because the IVC was almost completely obstructed and the hemodynamics were stable during cross-clamping of the IVC. Case 2: a 37-year-old female was transferred with a diagnosis of a giant metastatic liver tumor. A trisegmentectomy with resection of the invaded IVC and IVC replacement was performed while the abdominal aorta was cross-clamped to maintain the hemodynamics. Therefore, abdominal aortic cross-clamping was convenient to maintain the hemodynamics when the IVC replacement was performed during IVC cross-clamping.

## Introduction

Renal cell carcinoma (RCC) extending into the inferior vena cava (IVC) is observed in 4–19 % of RCC cases [1–9]. Tumor thrombectomy in the IVC with the tumor thrombus or nephrectomy improves the prognosis in patients with this condition, including those in whom the tumor thrombus extends into the vessel [5, 7, 10]. Both resection of the IVC and IVC replacement are required in cases where liver tumors have invaded the IVC [11, 12].

RCC and low-grade malignant tumors extending into the IVC have been resected without cardiopulmonary bypass (CPB) or venous bypass, by achieving hemodynamic stability with aortic cross-clamping [13, 14]. Good surgical results were obtained for different gastroenterological and urological diseases with IVC extension and IVC invasion. Therefore, this report presents the treatment strategies and the indications for IVC replacement, and reviews the pertinent literature.

## Case reports

### Case 1

**Fig. 1:**
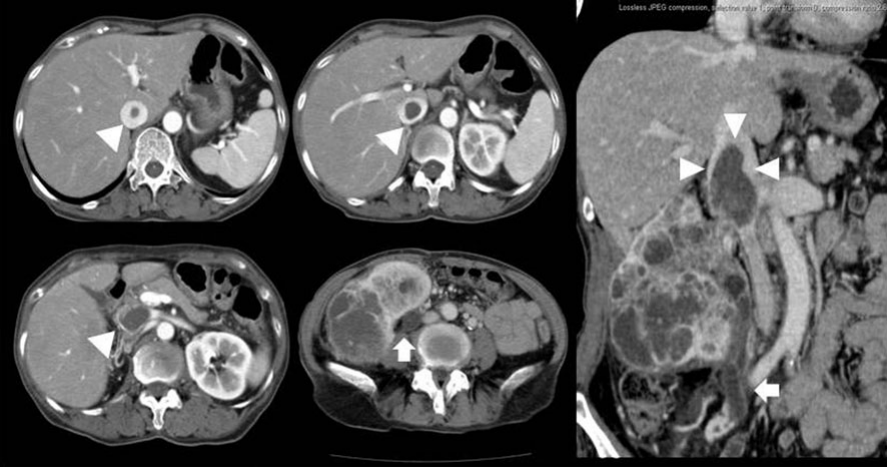
Computed tomography showed that there was a right renal cell carcinoma with a tumor thrombus extending to the site of the hepatic inferior vena cava (*arrowheads*) and that the right ureter also had a tumor thrombus (*arrows*)

A 68-year-old female was transferred to this institution with a diagnosis of right RCC with a tumor thrombus extending into the retrohepatic IVC (Fig. 1). No abnormalities were found on physical examination, and the results of routine laboratory tests and chest and abdominal Roentgen films were normal. General anesthesia was induced, and her cardiac function was monitored using transesophageal ultrasound. Laparotomy was performed. The right renal artery and vein were exposed. The segment of the suprahepatic IVC was exposed after minimal dissection of the pericardium and a plication was performed using 3-0 polypropylene to prevent extension into the right atrium. The IVC was exposed after the liver was mobilized. A nephrectomy and cavotomy with the tumor thrombus was performed for radical dissection, because the IVC was almost completely obstructed and the systemic blood pressure and hemodynamics were stable during cross-clamping of the infrahepatic IVC, without aortic cross-clamping. Finally, the left renal vein was ligated because the left renal vein stump pressure was 27 mmHg. The patient’s postoperative course was uneventful. She was discharged 33 days after the operation. Liver (S8) and bone (Th4) and lung metastases were diagnosed 3 months after the operation. Sunitinib malate (Sutent™) was administered because the primary tumor was interferon resistant. Zometa™ (zoledronate) treatment and 40 Gy of radiation were given for the bone metastasis. However, the patient died 11 months after the operation.

### Case 2

**Fig. 2:**
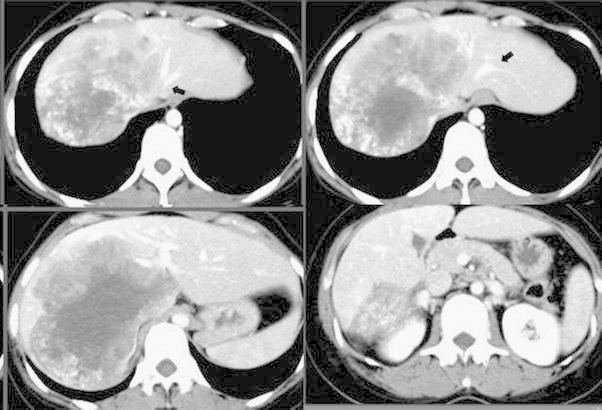
Computed tomography showed that there was a giant metastatic liver tumor that directly invaded the inferior vena cava and right and middle hepatic veins. However, it could not be determined whether the tumor had invaded the left hepatic vein (*arrows*)

A 37-year-old female underwent a radical operation for a right malignant parotid gland tumor (the pathological findings indicated that it was an adenoid cystic carcinoma) at another hospital. A giant metastatic liver tumor invading the IVC developed after the radical operation (Fig. 2), and the patient therefore underwent both transcatheter arterial chemoembolization and systemic chemotherapy using TS-1/CDDP combination chemotherapy (TS-1; 1 M tegafur-0.4 M gimestat-1 M otastat potassium, CDDP; cisplatin) four times, but the response evaluation criteria regarding the solid tumor status demonstrated no change. Therefore, she was transferred to this institution with a diagnosis of a giant metastatic liver tumor for right hepatic trisegmentectomy with resection of the invaded IVC. A physical examination revealed no abnormality, and the routine laboratory data were normal. The patient’s ICGR_15_ (retention rate) was 4.0 (normal 0–10). She was diagnosed to have lung metastasis but the tumor was a slow-growing adenoid cystic carcinoma. She was young and her cardiopulmonary function was normal. Therefore, a good prognosis could be expected if trisegmentectomy and resection of the IVC were performed.

**Fig. 3:**
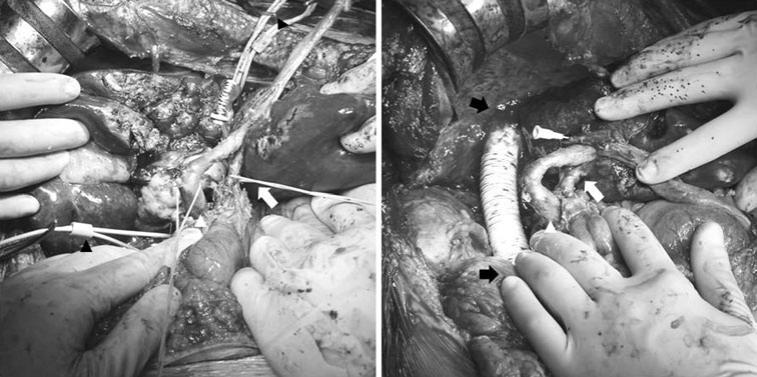
*Left* Heparin was administered, then the infrarenal abdominal aorta was clamped and the suprarenal inferior vena cava (IVC), suprahepatic IVC and left hepatic vein were clamped, as were the proper hepatic artery and portal vein. A right hepatic trisegmentectomy with resection of the invaded IVC was performed. *Black arrowheads* the suprahepatic and suprarenal IVC had been taped (*black arrowheads*). *White arrows* left hepatic artery, *white arrowheads* portal vein. *Right* An expansive polytetrafluoroethylene graft of 20 mm in diameter was interposed by 4-0 polypropylene continuous sutures while the infrarenal abdominal aorta was clamped. *Black arrows* the proximal and distal anastomosis, *white arrowhead* portal vein, *white arrow* left hepatic artery. The common bile duct had a tube inserted

Laparotomy was thus performed. The left hepatic vein, proper hepatic artery and portal vein were exposed. The suprarenal IVC and suprahepatic IVC were clamped before the right hepatic trisegmentectomy with resection of the invaded IVC, but the patient’s systemic systolic blood pressure fell below 80 mmHg during the IVC cross-clamping even after providing sufficient fluid replacement, so the IVC was immediately declamped. Infrarenal abdominal aortic cross-clamping was done without venovenous bypass to maintain the pressure at more than 100 mmHg, and to ensure hemodynamic stability after heparin was administered. The suprarenal IVC, suprahepatic IVC and left hepatic vein were clamped, and the proper hepatic artery and portal vein were also clamped. A right hepatic trisegmentectomy with resection of the invaded IVC was performed, because the left hepatic vein was not invaded by the tumor. The proximal IVC and a 20-mm expansive polytetrafluoroethylene (ePTFE) graft with removable rings were anastomosed using 4-0 polypropylene sutures. The suprahepatic IVC, left hepatic vein, proper hepatic artery and portal vein were declamped after clamping the infrahepatic IVC. Finally, the distal IVC and the graft were anastomosed using 4-0 polypropylene sutures, and the IVC was declamped and the infrarenal abdominal aorta was gradually declamped (Fig. 3). Biliary reconstruction was performed. Infrarenal abdominal aortic cross-clamping and the IVC were performed for 38 and 35 min, respectively. The left hepatic vein, proper hepatic artery and portal vein were clamped for 11 min. The patient’s postoperative course was uneventful. A partial resection of the liver (S3) and splenectomy were thereafter performed because metastasis to the residual liver and splenomegaly were diagnosed 10 months after the first operation. She was healthy at her last checkup 14 months after the first operation.

## Discussion

The surgical treatment of RCC with a tumor thrombus extending to the IVC is dependent on the disease site, thrombus extension level and the degree of IVC patency. Tumors are classified into four categories before surgery (level I: patients with tumor thrombus extending from the renal vein into the infrahepatic IVC for 1–2 cm and requiring only local control of the IVC for extraction, level II: patients with tumor thrombi that extend no further than the subhepatic IVC, level III: patients with thrombi that extend into the intrahepatic IVC or that extend to the suprahepatic IVC but not into the atrium, level IV: patients with intraatrial thrombi) according to the level of cephalad extension of the tumor thrombus into the IVC, as described by Naves and Zincke [1].

Level I patients with a cephalad extension of the tumor thrombus can have the segment of the infrahepatic IVC clamped without depending on any specific technique. The segment of the infrahepatic IVC can be clamped in level II patients as well, but careful mobilization and IVC exposure are important to prevent the development of a pulmonary embolism (PE). A temporary IVC filter should therefore be put in place on the day of surgery or the day before surgery, or an initial plication can be performed in patients with level II or III disease to prevent perioperative PE. A plication involves loosely ligating the IVC two or three times, using 3-0 polypropylene sutures in the IVC of the suprahepatic or retrohepatic segment to ensure that the IVC maintains its round shape. The plication or the temporary IVC filter is especially effective for preventing massive PE. An IVC filter can be placed cephalad to the thrombus or plication can be performed when the tumor extends to the portion just below the hepatic vein segment of the IVC. In addition, a plication can be performed when the tumor extends up to the suprahepatic vein segment of the IVC, or when there is not enough space to place an IVC filter even though the tumor might extend up to the portion below hepatic vein segment of the IVC before the liver can be mobilized [2, 14]. Cardiopulmonary bypass (CPB) without cardiac arrest is generally used in this institution, but other institutions have used CPB with cardiac arrest and deep hypothermia in patients with a level IV thrombus [4, 15, 16].

The systemic blood pressure sometimes falls to less than 80 mmHg during the cross-clamping of the IVC in patients with a patent IVC, because of the decrease in venous return. Therefore, sufficient fluid replacement and control of the hemodynamic circulation should be confirmed. The infrarenal abdominal aorta can be clamped partially or totally to maintain the blood pressure and hemodynamic circulation if the systemic blood pressure falls during cross-clamping of the IVC, and then tumor resection can be performed [13, 17]. Aortic cross-clamping and the Pringle maneuver are applied to minimize bleeding from the hepatic vein and to prevent hepatic congestion when the segment of the suprahepatic IVC is clamped. Venovenous bypass is sometimes used instead of aortic cross-clamping by other groups to maintain the hemodynamic stability [18–20].

The tumor thrombus of the RCC can be peeled off the IVC wall easily because the tumor thrombus extends into the IVC but does not usually invade the IVC [13]. However, it may be so difficult to exfoliate a tumor near the renal vein, and therefore both tumor thrombectomy with a wedge resection of the IVC need to be performed in order to completely resect a tumor [13]. Therefore, the excision of a tumor extending into the IVC and running sutures are usually performed in such cases without IVC resection. IVC resection is advisable when the IVC is occluded by a tumor thrombus. This technique may also be applied to patients with another tumor thrombus.

The left renal vein (RV) can be separated from the IVC in cases where there is an RCC originating from the right kidney that extends into the IVC, because the left RV has several branch veins (adrenal, ovarian, lumbar) draining into the hemiazygos system [13]. The renal function will be maintained when a left renal vein stump pressure of less than 35 mmHg is obtained, because the left RV can be divided if its stump pressure is about 50–60 cm of water (37–44 mmHg) or lower [21]. On the other hand, the connection between the right RV and caudal IVC should be preserved if the RCC from the left kidney extends into the IVC, or the right RV should be reconstructed to the caudal IVC because of the inadequate number of draining veins from the right kidney [13].

There are various surgical techniques, such as a partial IVC resection and direct closure or patch plasty, that can be used in cases of the direct invasion of liver tumors [11, 22–24]. However, it is sometimes necessary to interpose the IVC using an ePTFE graft with removable rings, although it is disadvantageous to replace the IVC using a graft because of the risk of the complications such as leakage of bile or pancreatic juice. The graft and the site of the anastomosis may be covered with the omentum. The superior mesenteric artery should be clamped or a venovenous shunt will be applied using a biopump or Anthron bypass tube™ (Toray, Tokyo, Japan) if the portal vein must be clamped for very long [25, 26].

IVC resection was performed to enhance the curability in case 1, but IVC replacement was not done, because the systemic blood pressure and hemodynamics were stable during cross-clamping of the infrahepatic IVC and the left RV stump pressure was 27 mmHg. Right hepatic trisegmentectomy with a resection of the invaded IVC was performed in case 2 to enhance the curability. It was necessary to perform the IVC reconstruction because the patient’s systemic systolic blood pressure fell below 80 mmHg during the IVC cross-clamping even after providing sufficient fluid replacement. Surgeons should decide whether to perform IVC replacement by considering intraoperative hemodynamics.

In conclusion, either partial or total abdominal aortic cross-clamping is considered to be a safe and simple technique and can be used to conveniently maintain hemodynamic stability without a shunt, even if the systemic blood pressure decreases during IVC cross-clamping when IVC replacement is performed for patients with gastroenterological, urological and gynecological diseases with IVC invasion or extension. Moreover, it is not necessary to reconstruct the IVC when the systemic blood pressure and hemodynamics are stable during cross-clamping the IVC without using a vasopressor after providing sufficient fluid replacement.
